# Entanglement Kinetics in Polymer Melts Are Chemically Specific

**DOI:** 10.1021/acsmacrolett.4c00092

**Published:** 2024-07-03

**Authors:** Benjamin E. Dolata, Marco A. Galvani Cunha, Thomas O’Connor, Austin Hopkins, Peter D. Olmsted

**Affiliations:** † Department of Physics and Institute for Soft Matter Synthesis & Metrology, Georgetown University, 3700 O St NW, Washington, D.C. 20007, United States; ‡ Department of Physics & Astronomy, University of Pennsylvania, Philadelphia, Pennsylvania 19104, United States; ¶ Department of Materials Science and Engineering, Carnegie-Mellon University, Pittsburgh, Pennsylvania 15213, United States; § Department of Physics, University of California Santa Barbara, Santa Barbara, California 93106, United States; ∥ Materials Science and Engineering Division, National Institute of Standards and Technology, Gaithersburg, Maryland 20899, United States

## Abstract

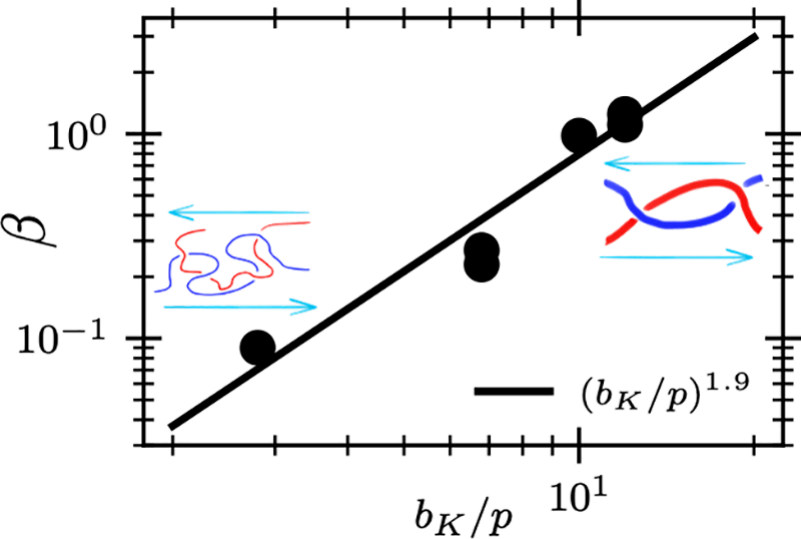

We investigate the universality of entanglement kinetics in polymer melts. We compare predictions of a recently developed constitutive equation for disentanglement to molecular dynamics simulations of both united-atom polyethylene and Kremer-Grest models for polymers in shear and extensional flow. We confirm that entanglements recover on the retraction time scale, rather than the reptation time scale. We find that the convective constraint release parameter β is independent of molecular weight, but that it increases with the ratio of Kuhn length *b*_*K*_ to packing length *p* as β ∼ (*b*_*K*_/*p*)^α^, with an exponent α = 1.9, which may suggest that disentanglement rate correlates with an increase in the tube diameter. These results may help shed light on which polymers are more likely to undergo shear banding.

The classical Doi–Edwards (DE) tube theory of entangled polymer melts approximates the many-body dynamics of a polymer molecule as a single-chain constrained within an effective tube arising from entanglements with the surrounding chains.^1^ This approximation leads to a dynamical equation for the polymer conformation (which encodes both polymer orientation and stretch) for a fixed number of entanglements. However, molecular dynamics simulations show that shear-induced disentanglement, often called convective constraint release (CCR),^2−9^ can occur for strong flows. In molecular models^10−15^ CCR is controlled by a parameter β, which represents the degree to which nonaffine retraction within a tube leads to the elimination of an entanglement. Experiments on apparent yield,^16^ step shear,^17^ repeated shear after short-time relaxation^18^ and steady shear^19^ have been interpreted in terms of disentanglement; and simulations have shown that the number of entanglements influences the properties of polymer materials, such as weld strength.^20−23^ Hence, there is a need for a physical understanding of the mechanisms that drive disentanglement.

The primary goal of this work is to elucidate how the CCR rate (encoded by the parameter β) depends on polymer chemistry. We study molecular dynamics simulations of three different model polymer “chemistries”, using our own simulations of flexible (F-KG) and semiflexible Kremer-Grest (SF-KG) bead spring polymers^24^ as well as data from three studies simulating united-atom polyethylene (UA-PE) by Nafar Sefiddashti et al.^5−8^ (Simulation details can be found in the Supporting Information, SI). The three molecules have different monomer densities and tube extensibilities, as quantified by the number of Kuhn segments per entanglement *N*_*eK*_, the number of chemical monomers per entanglement *N*_*e*_^mon^, and the characteristic ratio , where *R* is the end-to-end distance of a chain, *N*_mon_ is the number of monomers in the chain, and  is the length of a bond (Table 1).

**Table 1:** Parameters of the Simulated Polymers and Fitted CCR Parameters β^a^

Chemistry	*Z*_*k*,*eq*_	*N*_*eK*_	*N*_*e*_^mon^	*C*_∞_^*^	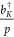	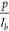	λ_max_	β_shear_	β^*B*^	β_ext_	β_ext_^*B*^
F-KG	12	40	86	1.9	2.8	1.9	6.3	0.09 ± 0.01	0.08	-	-
SF-KG-250	15	8.8	28	2.9	6.8	3.0	3.0	0.27 ± 0.02	-	-	-
SF-KG-500	31	8.8	28	2.9	6.8	3.0	3.0	0.23 ± 0.04	0.23	0.76	0.76
UA-PE^5^	9.0	6.0	65	8.2*	12	0.9	2.45	1.25 ± 0.11	2.0	-	-
UA-PE^6^	16.4	6.2	73	8.2	12	0.9	2.49	1.11 ± 0.08	-	-	-
UA-PE^7,8^	24.8	6.4	72	8.2	10	1.0	2.53	0.98 ± 0.16	2.0	-	-

a The packing length *p* has been expressed either in units of the Kuhn step *b*_*K*_ or the bond length  between particles (KG) or united atoms (UA-PE). The parametrization is discussed in the SI; for the KG chains, *Z*_*k*,*eq*_ was measured using the Z1 code,^26^ the characteristic ratio *C*_∞_ was obtained from Auhl et al.,^39^ all other quantities are taken from Everaers et al.^40^ The CCR parameters β_shear_, β_ext_ are obtained using Method A, while β^*B*^ and β_ext_^*B*^ are obtained using Method B. ^†^We use the relation between the packing length *p* and the bending modulus of KG chains given by Everaers et al.^40^ *The characteristic ratio *C*_∞_ for UA-PE chains is obtained from the data in Foteinopoulou et al.^41^ We use α = 0.5 in all cases. The F-KG simulations have chains of length *N*_mon_ = 500, while the SF-KG simulations have chains of lengths *N*_mon_ = 250, 500. Note that *Z* = *Z*_*k*_/2 is the number of “rheological” entanglements.

To characterize the CCR rate we compare the simulations to a thermodynamically consistent constitutive model for flow-induced disentanglement of a polymer melt recently developed by two of the authors.^15^ This model incorporates the Ianniruberto-Marrucci (IM) disentanglement mechanism,^12,13,25^ whereby entanglement removal is accelerated during flow (convective constraint release, or CCR). The CCR rate is characterized by a parameter β, which is roughly inversely proportional to the number of retraction events required to remove an entanglement, so that a smaller β requires more chain retraction events to effect entanglement removal.

Entanglements are inferred from the average number of “kinks” *Z*_*k*_ per chain, determined using the Z1 code,^26^ which implements a geometric algorithm to reduce the polymer chains to their primitive paths and identify kinks as the points where multiple primitive paths touch. The number of kinks *Z*_*k*_ is typically around twice the number of entanglements *Z* determined from the rheological tube.^2−4,27^ This discrepancy arises from spatial correlations among kinks, and is quantified by ζ_*Z*_ = *Z*_*k*_/*Z*, which may be understood as analogous to a characteristic ratio for the tube itself. We assume ζ_*Z*_ ≃ 2 throughout this work, so that *Z* = *Z*_*k*_/2 is the number of “rheological” entanglements.

The constitutive model couples the dynamics of the conformation tensor of the tube **A** (an average of the second moment of the tube segment vectors^15^), which encodes the deformation of the melt, to the entanglement ratio ν = ⟨*Z*_*k*_⟩/*Z*_*k*,*eq*_, which is the ratio of the current number of entanglements *Z* to the equilibrium value *Z*_*eq*_. The conformational dynamics are obtained by analogy with the Rolie-Poly model^28^ with an additional Giesekus term to describe a finite second normal-stress difference, parametrized here by α:^15^
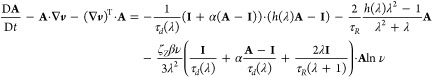
1a
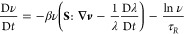
1b
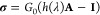
1cHere we approximate the orientation tensor of the tube as **S** = **A**/tr **A** and the polymer stretch as ; D/D*t* + ***v***·***∇*** is the material time derivative, ***v*** is the velocity field of the melt, **σ** is the stress tensor, τ_*R*_ and τ_*d*,*eq*_ are respectively the equilibrium Rouse and equilibrium reptation times, and τ_*d*,*eq*_ is computed from the Likhtman relation^29^ (see Dolata and Olmsted^15^ and the SI for details). The nonequilibrium reptation time
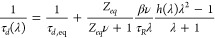
2arises self-consistently from the rate of entanglement removal at the chain ends, and accounts for thermal constraint release.^15,28^ The plateau modulus *G*_0_ = *Z*_*eq*_*nk*_B_*T* can be determined from the equilibrium number of entanglements (here *n* is the number density of polymer chains). The dimensionless FENE (finitely extensible nonlinear elastic)-spring constant is^30,31^
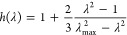
3where the maximum stretch is . The Giesekus parameter α ensures a nonzero second normal-stress difference.

The CCR rate β is the only fitting parameter; *Z*_*k*,*eq*_, τ_*R*_, τ_*d*,*eq*_, and λ_max_ can be measured or computed from linear response behavior, and α can be measured from weakly nonlinear rheology. In contrast to the assumptions of previous models,^12−14,25,32,33^ Dolata and Olmsted^15^ showed the entanglement ratio relaxes according to the Rouse time, rather than the reptation time. This is consistent with molecular dynamics simulations.^34^

The CCR parameter β of eq 1b was introduced by Marrucci^11^ to quantify the acceleration of the reptation rate due to the removal of entanglements in response to nonaffine motion of the primitive path (distinct from the chain itself). It was then incorporated by Ianniruberto and Marrucci^12^ into the dynamics for ν. In the GLaMM model CCR is determined self-consistently by equating the rate of removal of entanglements at the chain ends due to retraction with the rate of tube renewal in the tube interior,^10^ under the explicit assumptions that the tube persistence length is unchanged in flow and thus the number of entanglements is always proportional to the primitive path length, and that entanglements are two body interactions. The CCR dynamics postulated by Ianniruberto and Marrucci^12^ relaxes this requirement. Hawke et al.^14^ introduced the concept of “entanglement stripping” to describe the loss of entanglements due to relative motion of polymer chains caused by flow and chain retraction, in addition to the steady-state renewal and removal of entanglements incorporated in the GLaMM model.

We can estimate the dependence of β on polymer chemistry using physical arguments.The CCR parameter controls the rate of the release of topological constraints between neighboring chains. This suggests that β depends on Kuhn length *b*_*K*_, packing length *p*, and ⟨*R*^2^⟩, the three material properties that capture the packing geometry of simple monodisperse melts.^35−37^ Furthermore, β should be (approximately) independent of molecular weight (and hence ⟨*R*^2^⟩), because the Ianniruberto-Marrucci mechanism is independent of chain length. This suggests a functional form
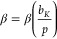
4Increasing *b*_*K*_/*p* will increase the ratio of the tube diameter *a* to the packing length,^38^ which suggests that β(*b*_*K*_/*p*) is an increasing function of its argument. Physically, disentanglement is easier when the confining tube is large compared to the characteristic separation between monomers.

We test the hypothesized form of eq 4 by estimating β from molecular dynamics simulations. There are two ways to use the solution of eq 1b to obtain β from simulated data of the steady state entanglement ratio ν(Wi_*R*_):

- A full calculation of eqs 1a (where **S** = **S**^*A*^ ≡ **A**/tr **A**) for the stress and entanglement dynamics, which requires a constitutive equation for the conformational tensor, can be compared with the simulated stress and entanglement number. In this method one should use model polymer parameters determined from simulations in the linear regime, aside from the anisotropy parameter α which is determined by the measured (simulated) normal stresses. This calculation simultaneously tests validity of stress predictions as well as disentanglement, and thus will fail if the constitutive relation is inaccurate.
- Alternatively, the equation of motion for the entanglement dynamics (eq 1b) has an exact solution in steady state that relates structure, flow rate, and disentanglement,^15^
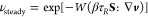
5where the Lambert *W* function *W*(*x*) obeys *We*^*W*^ = *x*. This can be fitted to simulation results for ν and **S** to find β. This relation holds for both extensional and shear flows. To compute the r.h.s. of (5) from molecular dynamics simulations one needs to calculate the orientation tensor. Unfortunately, it is almost impossible to accurately calculate the conformation tensor **A**, based on tube segments, from simulations, in order to compute **S**. Hence we compute the orientation tensor as
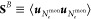
6where ***u***_*N*_*e*_^mon^_ is the unit vector pointing from a monomer (say *i*) to a second monomer located at a distance *N*_*e*_^mon^ from the first (i.e., *i* ± *N*_*e*_^mon^). This is the length scale over which the tube decorrelates, and is approximately equivalent to two kinks,^2−4,27^ and can be easily calculated from simulations. **S**^*B*^, like **A**, is a mean-field quantity, since the net tube orientation fluctuates more at the ends than in the middle.

Method A tests the full constitutive model, and the quality of the fit depends on the accuracy of the model and of the measured model inputs (τ_*d*,*eq*_, τ_*R*_, *N*_*eK*_, *etc*.), while Method B is less restrictive and tests the accuracy of the structural predictions of eq 1b for entanglement dynamics. Hence, Method B, which is independent of the assumed constitutive model, might be expected to give a more accurate measurement of β from simulations.

We first use Method A to estimate β for UA-PE and KG melts. The UA-PE simulation data were obtained from recent literature,^5−8^ while we performed simulations of the F-KG model using 500 monomers per chain, and SF-KG model using 250 or 500 monomers per chain. We calculate the entire coupled constitutive equation set (eq 1a) using the parameters in Table 1, which we then compare with the simulations. We first examine the influence of molecular weight on polymer disentanglement, based on the UA-PE simulations.^5−8^Figure 1 shows the simulated degree of disentanglement in steady shear flow as a function of Wi_*R*_ = γ̇τ_*R*_, where γ̇ is the shear rate. We determine β with a least-squares regression restricted to Wi_*R*_ ⩾ 0.1, and find β = 1.25 ± 0.11,1.11 ± 0.08, and β = 0.98 ± 0.16 for *Z*_*k*,*eq*_ = 9.0,^5^ 16.4,^6^ and 24.9,^7,8^ respectively, where the uncertainty represents 95% confidence. The small decrease in β with increasing *Z*_*k*,*eq*_ coincides with a slight increase in *N*_*eK*_ (cf. Table 1). However, all molecular weights are consistent with a single value β = 1.09 ± 0.08. Thus, molecular weight does not play an important role in entanglement kinetics.

**Figure 1:**
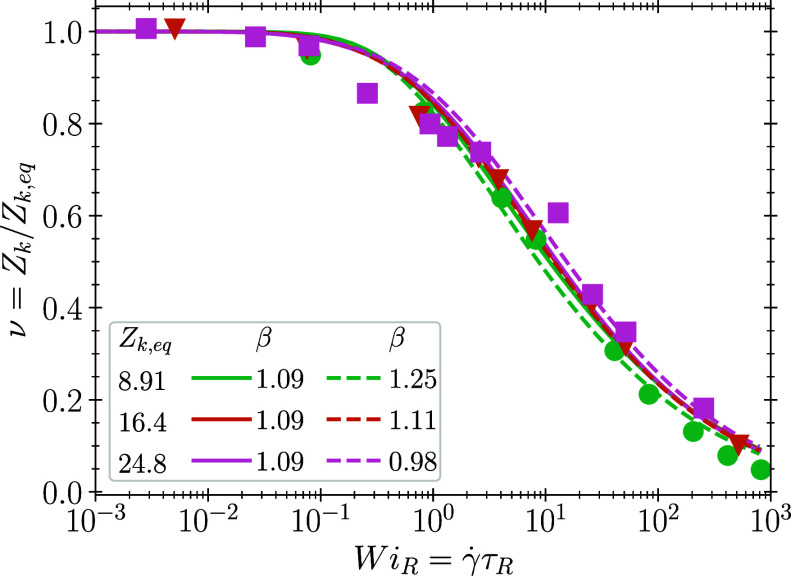
Comparison of our analytic model (lines, eq 5) with molecular simulation data (filled symbols) of disentanglement under steady state shear for three different molecular weights of UA-PE, for *Z*_*k*,*eq*_ = 9.0,^5^ 16.4,^6^ and 24.8.^7,8^ Solid lines are fits with the same β = 1.09, while dashed lines are best fit values.

We next compare disentanglement in steady-state shear between SF-KG and UA-PE simulations (Figure 2). The UA-PE melt is more disentangled than the SF-KG melt at a given Weissenberg number Wi_*R*_. Consequently, UA-PE displays a best fit β = 0.98 that is significantly larger than β = 0.23 for SF-KG. This implies that more retraction events are required to remove an entanglement in the SF-KG melt than in the UA-PE melt. Consistent with our hypothesized functional form in Eq. 4, stiffer polymers show a larger β (Table 1), and the data is approximately described by a power law (Figure 2(b)),
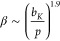
7Physically, this suggests that stiffer melts will disentangle more readily than more flexible melts. This power law may suggest that β increases with the ratio of tube diameter to the packing length, which scales as *a*/*p* ∼ (*b*_*K*_/*p*)^1/2^ in the semiflexible regime.^37,38^ We regard the observed power law as approximate, as we do not have enough data to rigorously justify the expression. In general, we expect the functional form of β(*b*_*K*_/*p*) to vary between the flexible, semiflexible, and stiff regimes in a manner similar to the plateau modulus^37,38^

**Figure 2:**
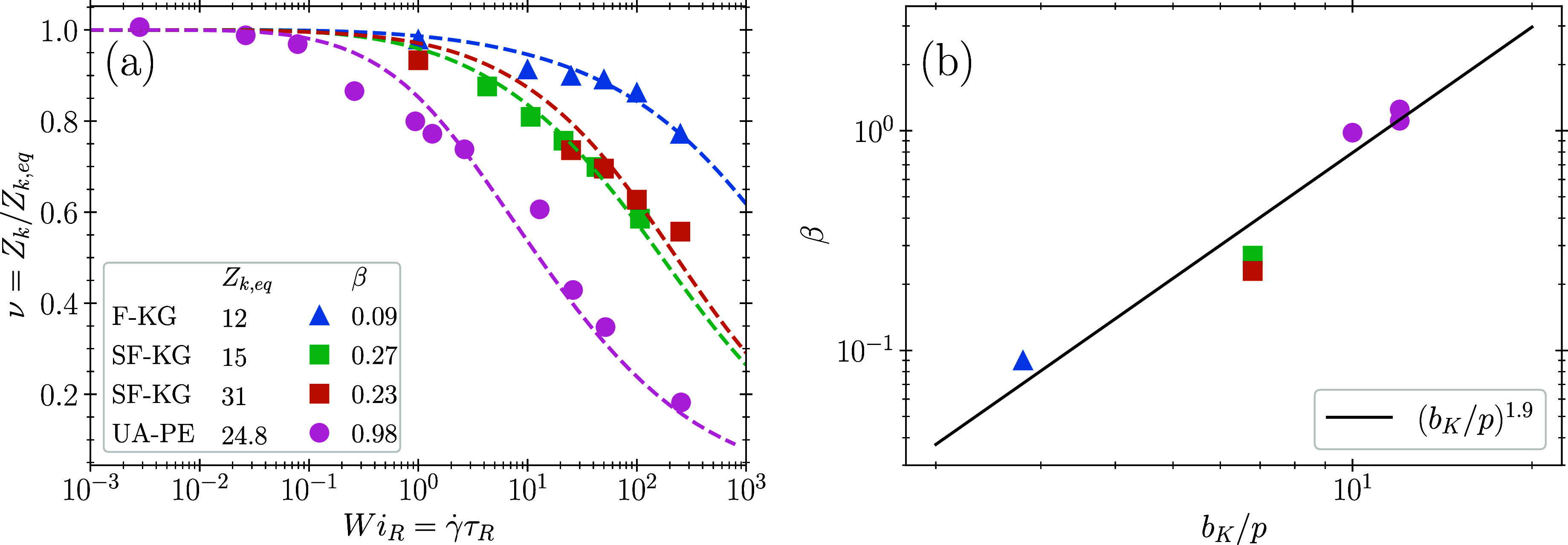
(a) Steady state disentanglement under shear flow for the SF-KG and UA-PE melts. Filled symbols are molecular dynamics simulations and solid lines are model fits to determine β using Method A. (b) CCR parameter β as a function of *b*_*K*_/*p*. Symbols represent the data from Table 1 and the black line is a power law fit.

We next compare Methods A and B in Figure 3 using the F-KG and SF-KG melts, for disentanglement under steady-state shear (S) at rate γ̇ and steady 3D uniaxial extension (E) at constant Hencky strain-rate ϵ̇. Methods A and B will necessarily produce almost identical fits in extensional flow because the molecules align nearly completely in the flow direction, so that τ_*R*_***∇****v*:**S** ≃ ϵ̇τ_*R*_.

**Figure 3:**
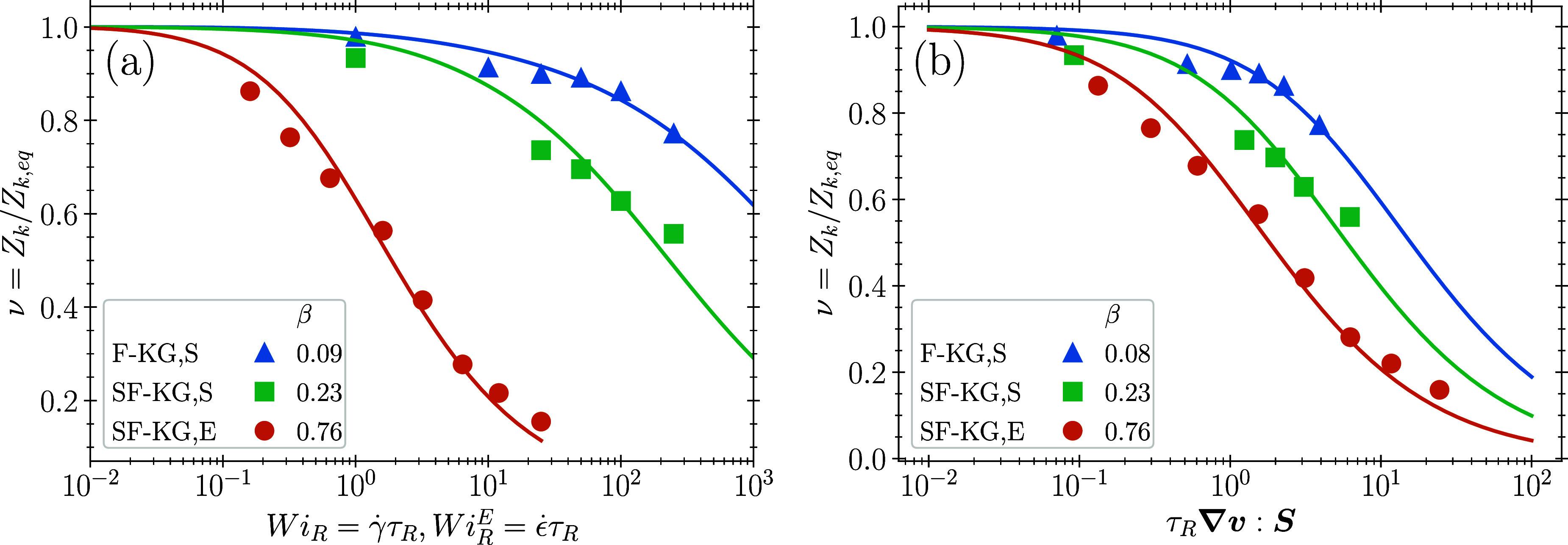
Disentanglement of the F-KG and SF-KG melts, both with 500 monomers, under shear (S) at constant shear rate γ̇ and extension (E) under constant extension rate ϵ̇. Filled symbols are simulation results, and solid lines are the best fit from Method A (a) and Method B (b).

The best fit value for β is larger for extension than for shear. The disagreement for different flow types indicates that the model^15^ is missing important physics, which could be due to several potential reasons. (1) This is a mean-field model, and assumes a spatially and dynamically homogeneous melt, whereas strong extensional flows can lead to dynamic and spatial heterogeneity, such as separation into domains of coiled (entangled) and stretched (unentangled) states.^42^ (2) Spatial heterogeneities in the entanglement distribution could lead to inhomogeneous chain deformation in extension analogous to that seen in polymer networks,^43^ which is not accounted for here. (3) Individual chains tumble in shear flow, while retraction dominates in extension, which leads to narrower distributions of conformations in extension and perhaps more accurate mean-field models. (4) Entanglements are treated as fixed, pointlike constraints, while in reality they fluctuate about some mean position, while under tension. Such fluctuating tensions are expected to have a tensorial (quadrupolar) nature that can be expected to differ between shear and extension. (5) Constraint release is assumed to be independent of the current state of the polymer conformation; however, the differences between polymer conformations in shear and extension could lead to multichain effects such that a given entanglement could be easier to remove in extension than in shear, which would lead to more rapid CCR and thus a larger β. (6) Entanglements are treated in a mean-field sense, while it has been shown that the distribution of entanglements along a chain is inhomogeneous following step elongation.^44^ The inhomogeneity may be expected to differ between shear and extension. Accounting for such inhomogeneity would require a multimode model, perhaps constructed in the manner of the GLaMM model.^10^

We could not apply Method B to the UA-PE data due to the absence of published data for **S**^*B*^. Instead, we approximate **S**^*B*^ using available data.^15^ For the simulations of *Z*_*k*_ = 9.0^4^ we approximate **S**^*B*^ ≃ **A**_*ee*_/tr **A**_*ee*_, where Baig et al.^4^ calculated **A**_*ee*_ = ⟨***R***_*ee*_***R***_*ee*_⟩ based on the polymer end-to-end vector ***R***_*ee*_. For the simulations of *Z*_*k*_ = 24.8^7,8^ we approximate **S**^*B*^ using the orientation tensor computed by Nafar Sefiddashti et al.^8^ from the unit vectors pointing between adjacent kinks from the Z1 code. Both cases lead to an approximate value of β^*B*^ = 2.0, which is larger than β^A^ = 1.09 ± 0.08 found using Method A. We know that both approximations will overestimate the tube segment orientation. The degree of orientation of the end-to-end vector exceeds the degree of orientation of shorter subsegments (except for the rare case of a fully stretched polymer), which would lead to a larger fitted value of β in order to match the simulations. Similarly, the orientation computed in the Z1 code exceeds that computed from the full chain because the Z1 code averages out fluctuations in entanglement positions, which again leads to a larger extracted value for β.

We now examine the kinetic predictions of our model by comparing numerical solutions of Eqs. 1a to the time-dependent re-entanglement in SF-KG chains following cessation of steady shear flow. We use the value β = 0.23 obtained from Method A. Results of molecular dynamics simulations are compared with model predictions in Figure 4 for three values of the Rouse Weissenberg number (Wi_*R*_ = 25,50,100). We observe good agreement between theory and simulation, with both displaying rapid re-entanglement by 3τ_*R*_, which confirms earlier suggestions that the melt re-entangles on the Rouse time.^15,34,45^ i.e., before the stress fully relaxes. Physically, the stretch and number of entanglements return to their equilibrium value on the Rouse time due to chain retraction in the still oriented tube. The subsequent relaxation of orientation requires renewal of the tube, which can only happen on the reptation time. This physical picture is consistent with molecular dynamics simulations,^34^ and contrasts with the interpretation of experiments that claim to determine entanglement dynamics from the rheology of interrupted shear.^18^ Further results for re-entanglement and steady state rheology are given in the Supporting Information.

**Figure 4:**
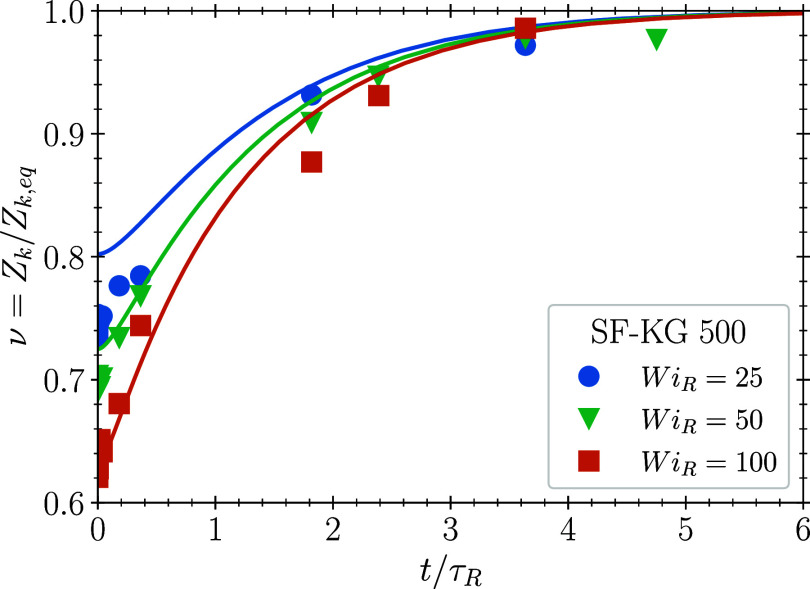
Re-entanglement following cessation of steady-shear. Filled circles are SF-KG 500 simulations, solid lines represent numerical solutions of Eq. 1a using the value β = 0.23 from fitting to the steady state shear data using Method A (Figure 2).

The fast re-entanglement within a time ∼ τ_*R*_ suggests that experiments that rely on stress measurements cannot unambiguously determine the state of entanglement. A common method that is claimed to measure reentanglement is to perform repeated shear startup experiments, where the second startup occurs a time τ_wait_ before the first experiment has fully relaxed after cessation of flow.^9,18,46^ For a short τ_wait_ < τ_*d*_ the stress overshoot during the second startup is very weak, which has been interpreted as a reduction of entanglements. It typically takes a relaxation time of order the reptation time, or even longer, for the second overshoot to reproduce that of a fully entangled melt.^9,18,46^ A large contribution to the decrease in the second stress overshoot in those experiments is probably due to the slow relaxation of chain alignment via reptation, rather than slow recovery of entanglements.^47^ Simulations by Galvani Cunha et al.^9^ showed that entanglements fully recover on the Rouse time. Moreover, these dynamics can also be crudely captured by the Rolie-Poly model, which assumes no change in entanglement number.^48^ As a consequence, the second stress overshoot should not be interpreted in terms of entanglement recovery. Similar behavior was observed in simulations of the welding of flow aligned layers, where the strength of re-entangled “healed” layers was degraded by residual alignment at the weld location, rather than by a loss of entanglements.^23^

In summary, we have extracted the CCR parameter β by fitting a constitutive equation and a structural model to steady-state simulations of KG and UA-PE melts. We have shown that β is independent of molecular weight but depends on polymer “chemistry”, with β scaling approximately as (*b*_*K*_/*p*)^1.9^, which implies that stiffer melts with larger tube diameters will disentangle more readily than more extensible melts. Furthermore, re-entanglement of the melt on the Rouse time was confirmed. Finally, we note that the magnitude of β is believed to influence shear banding behavior in entangled polymers.^49^ In the absence of CCR (β = 0) the shear stress has a maximum as a function of shear rate, which implies an instability to shear banding. Our results suggest that polymers with larger β, corresponding to larger *b*_*K*_/*p*, could be less susceptible to shear banding.^49^

## Supporting Information Available

Parameter determination, simulation details, and more comparison with the Ianniruberto-Marrucci model (PDF)
