# Supplementary material for: Entanglement Kinetics in Polymer Melts Are Chemically Specific
Source: ACS Macro Lett. 2024 Jul 3;13(7):896–902. doi: 10.1021/acsmacrolett.4c00092 (PMC11256759; doi:10.1021/acsmacrolett.4c00092)
Supplement: Supplementary file 1 — mz4c00092_si_001.pdf [file mz4c00092_si_001.pdf]

Supporting information for “Entanglement kinetics in polymer melts are chemically specific”

Benjamin E. Dolata,*,†,‖ Marco A. Galvani Cunha,‡ Thomas O'Connor,¶ Austin Hopkins,§ and Peter D. Olmsted*,†

†*Department of Physics and Institute for Soft Matter Synthesis & Metrology, Georgetown University, 3700 O St NW, Washington DC 20007, USA*

‡*Department of Physics & Astronomy, University of Pennsylvania, Philadelphia, PA 19104, USA*

¶*Department of Materials Science and Engineering, Carnegie-Mellon University, Pittsburgh, PA 15213, USA*

§*Department of Physics, University of California Santa Barbara, Santa Barbara, CA 93106, USA*

‖*Materials Science and Engineering Division, National Institute of Standards and Technology, Gaithersburg, MD 20899, USA*

E-mail: benjamin.dolata@nist.gov; pdo7@georgetown.edu

1 Table of quantities

Table S1: Table of symbols and quantities.

Symbol	Formula	Definition
N_{mon}		Number of united atoms per chain in the polyethylene melts or the number of bead per chain in the Kremer-Grest melts.
ℓ_b		Length of an individual bond.
b_K		Kuhn length of a polymer.
L		Primitive-path length of a polymer, as obtained from the Z1 code.
R_{max}		Maximum extension of a molecule with the bonds in the minimum energy configuration.
$\langle R^2 \rangle$		Mean square end-to-end length of a molecule.
Z_k		Number of topological entanglements, determined by counting the number of kinks in the Z1 code chain shrinking algorithms.[1]
$Z_{k,eq}$		The equilibrium number of topological entanglements.
Z or Z_{rheol}	$\begin{cases} \dfrac{\langle L \rangle^2}{\langle R^2 \rangle} \\ \dfrac{Z_{k,eq}+1}{2} \end{cases}$	The rheological number of entanglements.<br>Approximate relation obtained from Z1 code.
N_K	$\dfrac{R_{\text{max}}}{b_K}$	Number of Kuhn segments in the chain for Kremer-Grest chains.
N_K	$\dfrac{R^2_{\text{max}}}{\langle R^2 \rangle}$	Number of Kuhn segments in the chain for polyethylene chains.
N_{eK}	$\dfrac{N_K}{Z_{\text{rheol}}}$	Number of Kuhn segments in a *rheological* tube segment.
N_e^{mon}	$\dfrac{N_{\text{mon}}}{Z_{\text{rheol}}}$	Number of monomers in a rheological tube segment.
C_∞	$\dfrac{\langle R^2 \rangle}{\ell_b^2 (N_{\text{mon}}-1)}$	The characteristic ratio.
C_∞	$\dfrac{b_K}{\ell_b}$	The characteristic ratio for Kremer-Grests melts
λ	$\dfrac{\langle L \rangle}{L_{eq}}$	The (dimensionless) primitive path stretch.

Symbol	Formula	Definition
$\lambda_{\max}$	$\sqrt{N_{eK}}$	The maximum stretch. This quantity is independent of the current number of entanglements.[2]
$\mathbf{A}$		The conformation tensor defined as in Dolata and Olmsted[2] to be proportional to the stress and consistent with the stress-optical rule.
ν	$\dfrac{Z}{Z_{eq}}$	The entanglement ratio
$h(\lambda)$		The spring potential of a tube segment, given by the Cohen approximation to the inverse Langevin function.[3,4]
β		The CCR (convective constraint release) parameter, roughly inversely related to the number of retraction events necessary to remove an entanglement.
τ_e		The Rouse relaxation time for a chain the size of an entanglement strand. Computed using correlations from Everaers et al.[5] for the Kremer-Grest melts.
τ_R		The Rouse time, computed using $\tau_R = \tau_e Z_{\text{rheol}}^2$ for the Kramer-Grest melts. The united-atom polyethylene melts provide values of the Rouse time computed from the center-of-mass diffusivity using tube theory, and by identifying cross-over regimes in the segmental mean squared displacement.[6–8] We find that the tube theory values provide better fits for the polymer rheology, and use these values throughout.
τ_d	Eq. S3	The reptation (disengagement) time. computed from the Rouse time using the Likhtman[9] scaling relation.

2 Parameter Determination

The number of tube segments Z_{rheol} in the rheological tube sets the plateau modulus according to

$$G_0 = Z_{\text{rheol}} n k_{\text{B}} T, \tag{S1}$$

where n is the number density of polymer chains, k_{B} is Boltzmann constant, and T is the temperature. The two definitions of entanglements are related by

$$\zeta_Z = \frac{Z_{k,eq}}{Z_{\text{rheol}}} \tag{S2a}$$

$$\simeq \frac{2}{1 + 1/Z_{k,eq}} \tag{S2b}$$

$$\simeq 2, \tag{S2c}$$

which arises from comparing the topological number of kinks $Z_{k,eq}$ with the rheological value Z_{rheol}.[10–13] The equilibrium reptation time $\tau_{d,eq}$ can be computed from the rheological tube

segments as expressed in Eq. (S2a) through the Likhtman relation[9]

$$\frac{\tau_{d,eq}}{3Z_{\text{rheol}}} = \left(1 - \frac{3.38}{Z_{\text{rheol}}^{1/2}} + \frac{4.17}{Z_{\text{rheol}}} - \frac{1.55}{Z_{\text{rheol}}^{3/2}}\right)\tau_R. \tag{S3}$$

The Giesekus parameter[14]

$$\alpha \equiv -\lim_{\dot{\gamma}\to 0} 2\frac{\Psi_2}{\Psi_1} \tag{S4}$$

is defined by the ratio of the second and first normal stress coefficients in the limit of vanishing shear rate. This leaves the CCR parameter β as the only free parameter, since all others $(\alpha, N_{eK}, Z_{k,eq}, \tau_R)$ can be measured or computed.

Our prior work demonstrates that the best fit value of β will be insensitive to α over the range $0.2 < \alpha < 0.5$ (*c.f.* figure 6 of Dolata and Olmsted[2]). This range is consistent with experimentally determined values of α,[15] and is also consistent with the values of 4/7 and 2/7 predicted from tube theory with and without the independent alignment approximation (*c.f.* Eq. 7.205 of Doi and Edwards[16]) . As such, any physically reasonable value of α will yield approximately the same best-fit value of β. In this work, we use $\alpha = 0.5$ for all melts. We inferred this value from Nafar Sefiddashti et al.[8] for the longest united-atom polyethylene (UA-PE) melts using (S4), and assume that it holds regardless of molecular weight. We use value $\alpha = 0.5$ for the semi-flexible (FG-KG) and flexible (F-KG) Kremer-Grest melts because direct measurement of α required unfeasibly long computation time.

3 Simulation methods

All simulations of Kremer-Grest bead-spring models were performed using LAMMPS.[17] All melts simulated were at a number density of $n = 0.85/a^3$. Melts with $N = 500$ monomers per chain had $M = 368$ chains, while melts with $N = 250$ monomers per chain had $M = 736$ chains in total. Semiflexible chains of lengths $N = 250$ and $N = 500$; and flexible melts with $N = 500$ were simulated.

Monomers interact via a purely repulsive truncated Lennard-Jones (LJ) pair potential

$$U_{LJ} = \begin{cases} 4\epsilon\left[\left(\frac{a}{r}\right)^{12} - \left(\frac{a}{r}\right)^{6} + \frac{1}{4}\right] & r < r_c = 2^{1/6}a \\ 0 & r > r_c \end{cases} \tag{S5}$$

Relevant quantities measured in simulations are given in LJ units: m is the monomer mass, a is the monomer diameter, ϵ is the interaction energy, and time is given in units of $\tau = \sqrt{ma^2/\epsilon}$. The covalent bonds between beads in the same chain are modeled with the FENE potential

$$U_{\text{FENE}} = -0.5KR_0^2 \ln\left[1 - (r/R_0)^2\right], \tag{S6}$$

where $K = 30\epsilon/a^2$ and $R_0 = 1.5a$. For the semi-flexible chains, an additional angle bending potential is used, $U_{\text{bend}} = k_{\text{bend}}(1 - \cos\theta)$, where $k_{\text{bend}} = 1.5\epsilon$ and θ is the angle between successive bonds.

The KG simulations were carried out using the SLLOD algorithm as implemented in the

Large-scale Atomic/Molecular Massively Parallel Simulator (LAMMPS) and temperature was kept at a constant $T/\epsilon = 1$. SLLOD works by imposing a uniform shear velocity profile across the simulation box, and therefore is not able to accurately describe inhomogeneous behavior such as shear banding. During shear, temperature was controlled by a Nosé-Hoover thermostat with a damping time of 10τ. During relaxation after cessation of shear, temperature was controlled by a Langevin thermostat that subtracts the remaining velocity profile from the shear stage during the early stages of relaxation, before adjusting the temperature. Different protocols during cessation (Nosé-Hoover thermostatting, not removing the velocity profile and thermostatting only velocities perpendicular to shear plane) were tested and found to not significantly alter the results for the rates studied.

Extensional flow simulations utilize generalized Kraynik-Reinelt boundary conditions to avoid the problem of the simulation box becoming too narrow at large strains.[18–20] The temperature control and damping time were the same as those used in the shear simulation.

'Kinks' were calculated using the Z1 code,[?] which identifies topological constraints between polymers and outputs the primitive path. This method differs from the traditional PPA because it is not based on molecular dynamics, but on a series of topological moves that reduce the chains to their topological constraints. A more recent version of the code[21] is available, but was not used in these calculations. At equilibrium, the number of kinks Z_k is roughly proportional to the number of rheological entanglements, $Z_k \simeq 2Z_{\text{rheo}}$.

Our methods were equivalent to the methods employed in the UA-PE simulations.[6–8] The UA-PE simulations used the SKS (Siepmann-Karaborni-Smit) potential[22] and were performed in LAMMPS using the SLLOD algorithm and temperature was controlled at $T = 450\,\text{K}$ using a Nosé-Hoover thermostat. The number of entanglements was computed using the Z1 code.

4 Comparison with the Ianniruberto-Marruci Model

4.1 Cessation of a Steady Shear Flow

We compare the results of our model and the Ianniruberto-Marruci (IM) model following the cessation of a steady shear flow. Ianniruberto and Marrucci[23,24] calculated the stretch dynamics according to

$$\frac{\partial \lambda}{\partial t} = -\lambda \mathbf{S} : \boldsymbol{\nabla v} - \frac{\lambda - \nu^{1/2}}{\tau_R}. \tag{S7}$$

where the orientation tensor $\mathbf{S}$ was calculated using the Doi-Edwards model, with the reptation rate replaced by its modification due to CCR,

$$\frac{1}{\tau_d(\beta)} = \frac{1}{\tau_{d,eq}} + \beta\, \nabla \mathbf{v} : \mathbf{S}, \tag{S8}$$

and with the affine deformation computed using a Seth tensor. We will compute the entanglement dynamics in the IM model using both their original expression where entanglements recover on the reptation time and a modified expression consistent with the Rouse relaxation

of our model:

$$\text{IM Original:} \qquad \frac{\partial \nu}{\partial t} = -\beta\nu\left(\nabla \mathbf{v} : \mathbf{S} - \frac{d\ln\lambda}{dt}\right) - \frac{\nu - 1}{\tau_d}, \tag{S9a}$$

$$\text{IM Modified:} \qquad \frac{\partial \nu}{\partial t} = -\beta\nu\left(\nabla \mathbf{v} : \mathbf{S} - \frac{d\ln\lambda}{dt}\right) - \frac{\ln\nu}{\tau_R}, \tag{S9b}$$

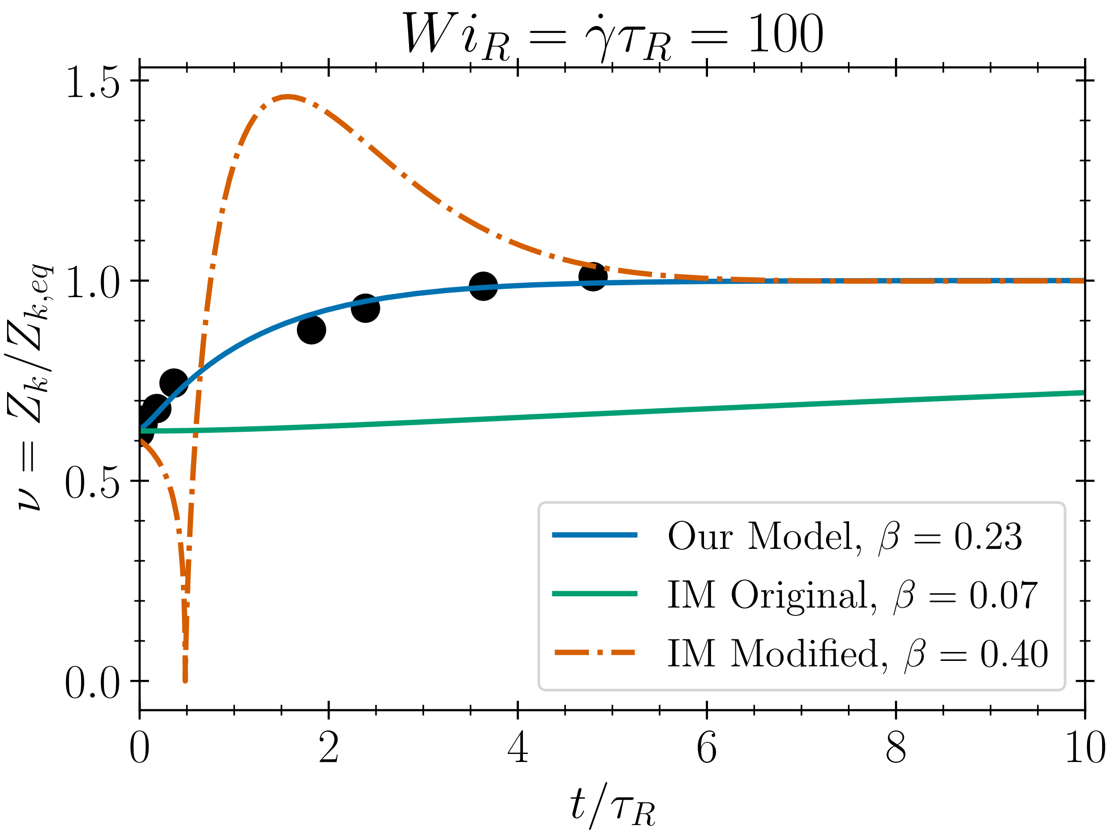

Figure S1: Comparison between our model and the IM model for re-entanglement of the SF-KG melt with 500 monomers following cessation of a steady shear. Lines represent theoretical calculations and symbols represent simulation results.

In figure S1, we compare simulated results for re-entanglement of the SF-KG 500 melt following cessation of a flow at $Wi_R = 100$; in the IM-model, we choose β to give best fit of ν in steady state flow. We find that our model describes the re-entanglement of the melt accurately, while the IM model with re-entanglement on the reptation time re-entangles too slowly. The IM model with re-entanglement on the Rouse time is unstable; the number of entanglements initially drops to zero, grows to a value above equilibrium, and then decays towards equilibrium on the Rouse time. This behavior arises from the form of the modified

IM equations during retraction after the cessation of the flow:

$$\frac{\partial \lambda}{\partial t} = -\frac{\lambda - \nu^{1/2}}{\tau_R}, \tag{S10a}$$
$$\frac{\partial \nu}{\partial t} = \frac{\beta\nu}{\lambda}\frac{\partial \lambda}{\partial t} - \frac{\ln \nu}{\tau_R}. \tag{S10b}$$

Here, the initially stretched primitive path will contract, removing entanglements at the chain ends. This disentanglement increases $\partial\lambda/\partial t$ in (S10a), causing further disentanglement. If β is sufficiently large, this continues until the number of entanglements and $\partial\lambda/\partial t$ approaches zero, giving rise to a strong thermodynamic driving force that increases the number of entanglements. Further refinements of the IM model that introduced a multimode version of the stretch relaxation equation[25] may resolve the instability observed during relaxation.

Essentially, the instability is driven by the assumption that the stretch relaxes to a locally-equilibrated tube of length $\nu^{1/2}$. This assumption, while reasonable on its face, is contradicted by the observation that the relaxation of stretch under the addition and removal of entanglements is asymmetric due to the fast relaxation of Kuhn segments following entanglement removal.[26] This asymmetry implies that the addition and removal of entanglements is an inherently dissipative process, and so $\lambda = \nu^{1/2}$ cannot be regarded as a local equilibrium for the stretch. Furthermore, the couplings between ν and λ in Eq. (S10a) are forbidden by Onsager-Casimir reciprocity.[2] The considerations highlight the importance of incorporating thermodynamic constraints in rheological constitutive equations.

4.2 Steady-State Rheological Predictions

Fig. S2 compares predictions of our model for the shear viscosity with the shear viscosity of the longest UA-PE chains,[8] for $\beta = 0.2, 0.6, 1.0$, where $\beta = 1.0$ provided the best fit to shear-induced disentanglement. We see good agreement for all β between the model predictions (solid lines) for the shear viscosity and the simulations (filled circles) over the entire range of Wi_R, with discrepancies increasing as the shear rate approaches the inverse entanglement time τ_e^{-1}. On these timescales, simulations of UA-PE have shown that polymer molecules undergo cycles of retraction and extension[27] that are not described by our model, which may contribute to an over-prediction of the viscosity. The viscosity and first normal-stress coefficient are fairly insensitive to the value of the CCR parameter β, which suggests that the shear viscosity is a weak discriminator for β. Interestingly, the best fit of β obtained from the UA-PE method via Method A also provides the best fit for the thinning of the second normal stress coefficient. This suggests that Ψ_2 could be a better discriminant for β than the other rheometric coefficients.

5 Disclaimer

Certain commercial or open-source software are identified in this paper in order to specify the methodology adequately. Such identification is not intended to imply recommendation or endorsement of any product or service by NIST, nor is it intended to imply that the software identified are necessarily the best available for the purpose.

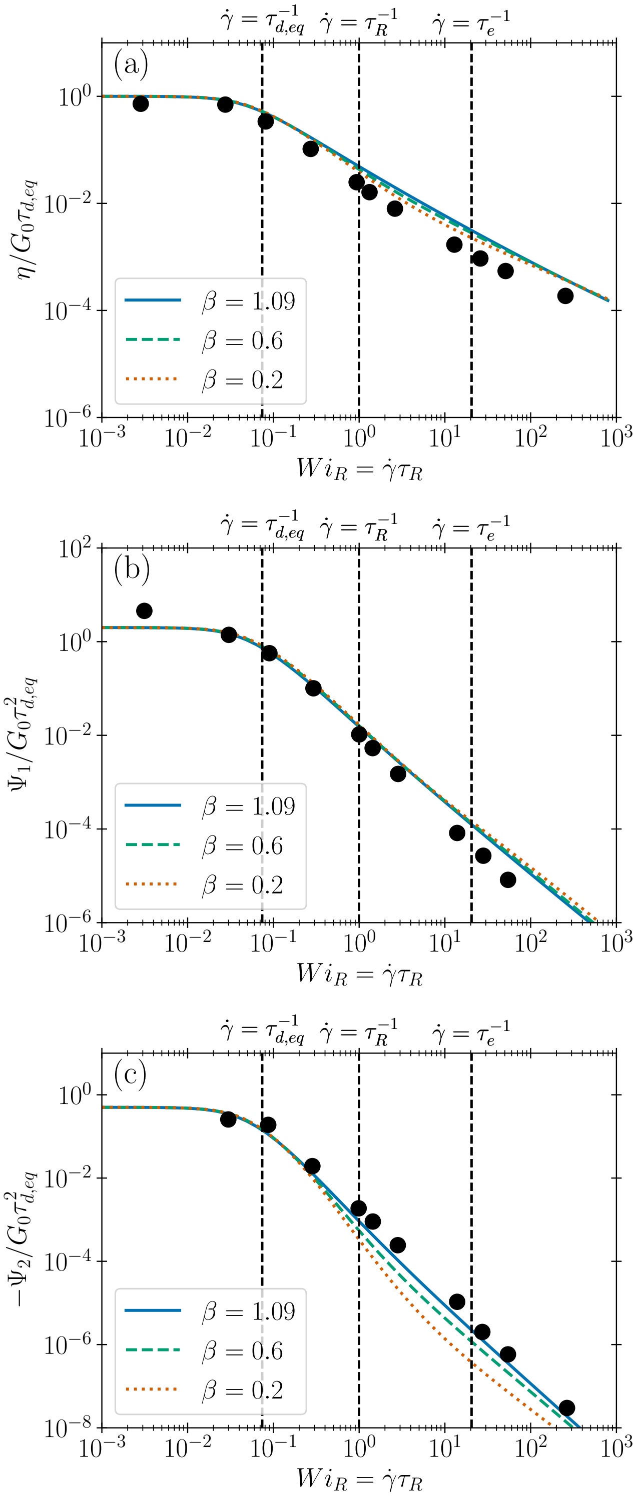

Figure S2: (a) Steady-shear viscosity η, (b) first normal stress coefficient Ψ_1, and (c) second normal stress coefficient Ψ_2 as a function of Wi_R. Filled circles are UA-PE with $Z_{k,eq} = 24.8$,[8] solid lines represent our model.

References

(1) Shanbhag, S.; Kröger, M. Primitive path networks generated by annealing and geometrical methods: Insights into differences. Macromolecules **2007**, 40, 2897–2903.

(2) Dolata, B. E.; Olmsted, P. D. A thermodynamically consistent constitutive equation describing polymer disentanglement under flow. Journal of Rheology **2023**, 67, 269–292.

(3) Cohen, A. A Padé approximant to the inverse Langevin function. Rheological Acta **1991**, 30, 270–273.

(4) Stephanou, P. S.; Baig, C.; Mavrantzas, V. G. A generalized differential constitutive equation for polymer melts based on principles of nonequilibrium thermodynamics. Journal of Rheology **2009**, 53, 309–337.

(5) Everaers, R.; Karimi-Varzaneh, H. A.; Fleck, F.; Hojdis, N.; Svaneborg, C. Kremer–Grest models for commodity polymer melts: linking theory, experiment, and simulation at the Kuhn scale. Macromolecules **2020**, 53, 1901–1916.

(6) Nafar Sefiddashti, M. H.; Edwards, B. J.; Khomami, B. Individual chain dynamics of a polyethylene melt undergoing steady shear flow. Journal of Rheology **2015**, 59, 119–153.

(7) Nafar Sefiddashti, M. H.; Edwards, B. J.; Khomami, B. Steady shearing flow of a moderately entangled polyethylene liquid. Journal of Rheology **2016**, 60, 1227–1244.

(8) Nafar Sefiddashti, M. H.; Edwards, B. J.; Khomami, B. Elucidating the Molecular Rheology of Entangled Polymeric Fluids via Comparison of Atomistic Simulations and Model Predictions. Macromolecules **2019**, 52, 8124–8143.

(9) Likhtman, A. E.; McLeish, T. C. B. Quantitative theory for linear dynamics of linear entangled polymers. Macromolecules **2002**, 35, 6332–6343.

(10) Masubuchi, Y.; Ianniruberto, G.; Greco, F.; Marrucci, G. Entanglement molecular weight and frequency response of sliplink networks. The Journal of Chemical Physics **2003**, 119, 6925–6930.

(11) Tzoumanekas, C.; Theodorou, D. N. Topological analysis of linear polymer melts: a statistical approach. Macromolecules **2006**, 39, 4592–4604.

(12) Foteinopoulou, K.; Karayiannis, N. C.; Mavrantzas, V. G.; Kröger, M. Primitive path identification and entanglement statistics in polymer melts: Results from direct topological analysis on atomistic polyethylene models. Macromolecules **2006**, 39, 4207–4216.

(13) Baig, C.; Mavrantzas, V. G.; Kröger, M. Flow effects on melt structure and entanglement network of linear polymers: Results from a nonequilibrium molecular dynamics simulation study of a polyethylene melt in steady shear. Macromolecules **2010**, 43, 6886–6902.

(14) Giesekus, H. A simple constitutive equation for polymer fluids based on the concept of deformation-dependent tensorial mobility. Journal of Non-Newtonian Fluid Mechanics **1982**, 11, 69–109.

(15) Maklad, O.; Poole, R. J. A review of the second normal-stress difference; its importance in various flows, measurement techniques, results for various complex fluids and theoretical predictions. Journal of Non-Newtonian Fluid Mechanics **2021**, 104522.

(16) Doi, M.; Edwards, S. F. The Theory of Polymer Dynamics; Oxford University Press, 1988.

(17) Thompson, A. P.; Aktulga, H. M.; Berger, R.; Bolintineanu, D. S.; Brown, W. M.; Crozier, P. S.; in't Veld, P. J.; Kohlmeyer, A.; Moore, S. G.; Nguyen, T. D.; others LAMMPS-a flexible simulation tool for particle-based materials modeling at the atomic, meso, and continuum scales. Computer Physics Communications **2022**, 271, 108171.

(18) Dobson, M. Periodic Boundary Conditions for Long-Time Nonequilibrium Molecular Dynamics Simulations of Incompressible Flows. The Journal of Chemical Physics **2014**, 141, 184103.

(19) Nicholson, D. A.; Rutledge, G. C. Molecular Simulation of Flow-Enhanced Nucleation in n-Eicosane Melts under Steady Shear and Uniaxial Extension. The Journal of Chemical Physics **2016**, 145, 244903.

(20) O'Connor, T. C.; Hopkins, A.; Robbins, M. O. Stress relaxation in highly oriented melts of entangled polymers. Macromolecules **2019**, 52, 8540–8550.

(21) Kröger, M.; Dietz, J. D.; Hoy, R. S.; Luap, C. The Z1+ package: Shortest multiple disconnected path for the analysis of entanglements in macromolecular systems. Computer Physics Communications **2023**, 283, 108567.

(22) Siepmann, J. I.; Karaborni, S.; Smit, B. Simulating the critical behaviour of complex fluids. Nature **1993**, 365, 330–332.

(23) Ianniruberto, G.; Marrucci, G. Convective constraint release (CCR) revisited. Journal of Rheology **2014**, 58, 89–102.

(24) Ianniruberto, G.; Marrucci, G. Erratum: "Convective constraint release (CCR) revisited" [*J. Rheol.* 58, 89-102 (2014)]. Journal of Rheology **2014**, 58, 1083–1083.

(25) Ianniruberto, G. Quantitative appraisal of a new CCR model for entangled linear polymers. Journal of Rheology **2015**, 59, 211–235.

(26) Hawke, L. G. D.; Huang, Q.; Hassager, O.; Read, D. J. Modifying the pom-pom model for extensional viscosity overshoots. Journal of Rheology **2015**, 59, 995–1017.

(27) Nafar Sefiddashti, M. H.; Edwards, B. J.; Khomami, B. Individual molecular dynamics of an entangled polyethylene melt undergoing steady shear flow: steady-state and transient dynamics. Polymers **2019**, 11, 476.
